# Recurrent Pregnancy Loss and Thrombophilia

**DOI:** 10.4021/jocmr2010.02.260w

**Published:** 2010-02-26

**Authors:** Maristella D’Uva, Pierpaolo Di Micco, Ida Strina, Giuseppe De Placido

**Affiliations:** a Department of Obstetrics and Gynecology and Human Reproduction, Federico II University of Naples, Naples, Italy; b Internal Medicine Division, Buonconsiglio Fatebenefratelli Hospital of Naples, Naples, Italy

## Abstract

Emerging data seem to be available also on the role of active thromboprophylaxis with heparin and pregnancy outcome. In the last decades we found many data concerning the association between a hypercoagulable state and its causes and adverse pregnancy outcome, in particular recurrent pregnancy loss (RPL). First studies which focused on the association between thrombophilia and RPL underlined the role of reduced clotting inhibitors and RPL, and subsequent studies underlined a pathogenetic role of gene variant associated to hypercoagulable state in the occurrence of RPL. On the other hand, acquired thrombophilic abnormalities as antiphipsholipid syndrome are a well known cause of RPL and should be considered for a screening. These data are relevant because recent studies suggested a role of an extensive thromprophilaxis in women with RPL that should be addressed only in case of known thrombophilia and high risk of venous thromboembolism.

**Keywords:**

Thrombophilia; Recurrent pregnancy loss; Factor V Leiden; Hyperhomocysteinemia; Antiphospholipid antibodies; PAI 4G\4G

## Introduction

Recurrent pregnancy loss (RPL) represents a major health problem with two to three or more losses in up to 5% of women of reproductive age and is actually one of the most common causes of female sterility [1]. Several reports identify inherited predisposition to thrombophilia as one of the main causes of RPL in particular if several diseases potentially responsible of RPL have been already excluded such as endocrine diseases (such as ovarian dysfunction, anovulation, hypopituitarism and diabetes), uterine malformation, genetic alterations (for example, chromosomal aberrations), inflammatory diseases (in particular systemic lupus erythematosus) and infectious diseases [2-5]. From a pathological point of view, women affected by thrombophilia show during their pregnancy a hypercoagulable state that is already increased during pregnancy, which may impair placental flow and then its function and fetal growth and may predispose to develop venous thrombosis [6].

During pregnancy, in fact, we may observe many changes in the haemostatic balance with a trend toward thrombophilia in order to be prompt for the haemostatic challenge of delivery [2, 6-7]. Thus, pregnancy is a condition associated to thrombophilia per se and for this reason it is associated with the increase of several clotting factors (namely factor VIII, vWF, fibrinogen and factor VII) [7]. Moreover, also other markers of a hypercoagulable state are increased during pregnancy, such as D-dimer and/or prothrombin fragment 1+2 [7,8]. For this reason we may observe episodes of venous thromboembolism (VTE) during pregnancy [9]. Moreover, women carrying further thrombotic risk factors such as inherited thrombophilia show an additionally increased risk of thrombotic events during pregnancy such as venous thromboembolism and/or abortion [10].

VTE and pulmonary embolism (PE), in fact, continue to be a leading cause of maternal death during pregnancy or postpartum and may cause significant morbidity of pregnant women.

The aim of the review is to focus fundamental clinical aspect of thrombophilias in the occurrence of RPL.

## Inherited Thrombophilia and Pregnancy Loss

Thrombophilia has been identified as one of the main causes of RPL with a percentage of until 40%, in particular early RPL [11]. Although several studies on this topic are available in the literature to confirm this trend, rates of thrombophilia seem to differ from study to study because of different inclusion criteria and different ethnic backgrounds of the selected patients [12]. In this clinical setting we may differentiate inherited thrombophilia, acquired thrombophilia and combined thrombophilia [13-14].

Inherited thrombophilia may be due to deficiency of clotting inhibitors or to gene variants leading to a hypercoagulable tendency. Gene variants frequently associated with RPL are prothrombin A20210G and/or factor V Leiden. Prothrombin A20210G has been identified as a risk factor for pregnancy loss in several studies and has been associated mainly to early RPL [15-19]. On the other hand, factor V Leiden, which is responsible for more than 75% of inherited activated protein C resistance, is the more common inherited thrombotic risk factor associated to RPL [20-22]. In particular, a case control study by Ridker et al. has reported an increased prevalence of FVL in women with RPL, while other studies revealed a strong relationship between FVL and early RPL [23]. FVL has been identified as a risk factor also for late RPL [24]. Also deficiency of clotting inhibitors, such as protein S, protein C and/or antithrombin, has been clearly associated to RPL since 1996 [25,26].

In the latest years an emerging role has been suggested and underlined also for the PAI-1 4G\5G gene variant that may be associated to hypofibrinolysis and so to hypercogulable state. Several reports underlined the association between 4G\4G genotype of PAI-1 and RPL [27,28] and this association seems to be relevant if anamnestic VTE is also present [29]. Yet more detailed data on large based population are needed in next years.

## Hyperhomocysteinemia

A pathogenetic role of hyperhomocysteinemia (HHCY) in RPL has been underlined by several reports on this topic, but data available in the literature are actually not univocal. Several authors reported increasing evidences for the relationship between HHCY, methylenetetrahydrofolate reductase gene polymorphism C677T (MTHFR C677T) and RPL, in particular early RPL [25,30-32]. On the other hand, further authors found a negative association between HHCY and early RPL [33-35].

## Acquired Thrombophilia

Several authors underlined the role of the antiphospholipid syndrome (APS) in the pathophysiology of RPL [36-48]. To confirm this point, actually adverse pregnancy outcome is considered as one of the diagnostic criteria of APS (Table 1) according to the guidelines of the International Society of Thrombosis and Haemostasis and the American Rheumatology Association [49,50]. During APS, a large variety of autoantibodies also toward clotting factors, such as factor XII, has been found [51,52]. However, a clear explanation of all involved processes on the roles of antiphospholipid antibodies and of autoantibodies toward clotting factors is still matter of discussion.

**Table 1:** Diagnostic Criteria to Detect Antiphospholipid Syndrome

Clinical Criteria *(Vascular thrombosis of arterial and/or venous vessels in any tissue or organ)* *Pregnancy morbidity*	Laboratory Criteria
	
One or more unexplained deaths of a morphologically normal fetus at or beyond the 10th week of gestation	Lupus anticoagulant present in plasma, on two or more occasions at least 12 weeks apart, detected according to the International Society of Thrombosis and Haemostasis (ISTH)
	
One or more unexplained deaths of a morphologically normal neonate before the 34th week of gestation because of eclampsia or severe eclampsia or placental insufficiency	Anticardiolipin antibody (aCL) of IgG and/or IgM isotype in serum or in plasma present in medium or high titer on two or more occasions at least 12 weeks apart
	
Three or more unexplained consecutive spontaneous abortions before the 10th week of gestation	Anti-β_2_ glycoprotein-I antibody of IgG and/or IgM isotype in serum or in plasma present on two or more occasion at least 12 weeks apart

For more details we suggest to consult Miyakis S, et al. J Thromb Haemost. 2006;4:295-306 [50].

On the other hand, new evidence seems to be available for the role of increased maternal plasma levels of clotting factor VIII and the risk of RPL [53]. Furthermore, acquired activated protein C resistance, which is not associated with the presence of FVL, has been described in several women with RPL, but also in this case, not all involved mechanisms are known [54].

## Combined Thrombophilia

Combined thrombophilia, namely inherited thrombophilia associated with acquired thrombophilia or more than one inherited thrombophilic defect, has also been identified as a cause of RPL, but its real frequency is not clear. Several studies in the latest years identified combined thrombophilic defects in women with RPL both early RPL and late RPL [10,28,55].

## Conclusions

Active surveillance of women referred to gynecological centers for RPL should be supported by thrombophilia screening. This approach may be helpful to fight this major health problem that involves up to 5% of women of reproductive age by an appropriate antithrombotic treatment. Inherited and/or acquired thrombophilia has been diagnosed in 50% to 65% of women with history of unexplained RPL and in nearly 20% of women with RPL with age of more than 35 years [56].

This gynecological and clinical aspect may be considered in particular if anamnesis reveals the presence of personal and\or familial trend to develop thrombotic disorders in particular VTE. So an appropriate clinical evaluation focused on diagnosis and therapy of RPL should also consider thrombophilic defects.
